# The FATZO mouse, a next generation model of type 2 diabetes, develops NAFLD and NASH when fed a Western diet supplemented with fructose

**DOI:** 10.1186/s12876-019-0958-4

**Published:** 2019-03-18

**Authors:** Gao Sun, Charles V. Jackson, Karen Zimmerman, Li-Kun Zhang, Courtney M. Finnearty, George E. Sandusky, Guodong Zhang, Richard G. Peterson, Yi-Xin (Jim) Wang

**Affiliations:** 1 Crown Bioscience Taicang Inc, Taicang, China; 2 Crown Bioscience Indiana Inc, Indianapolis, USA; 3 Department of Pathology and Laboratory Medicine, Indiana University School of Medicine, Indianapolis, Indiana USA

**Keywords:** Metabolic disease, Liver disease, NAFLD, NASH, FATZO mouse

## Abstract

**Background:**

Metabolic disorders such as insulin resistance, obesity, and hyperglycemia are prominent risk factors for the development of non-alcoholic fatty liver disease (NAFLD)/steatohepatitis (NASH). Dietary rodent models employ high fat, high cholesterol, high fructose, methionine/choline deficient diets or combinations of these to induce NAFLD/NASH. The FATZO mice spontaneously develop the above metabolic disorders and type 2 diabetes (T2D) when fed with a normal chow diet. The aim of the present study was to determine if FATZO mice fed a high fat and fructose diet would exacerbate the progression of NAFLD/NASH.

**Methods:**

Male FATZO mice at the age of 8 weeks were fed with high fat Western diet (D12079B) supplemented with 5% fructose in the drinking water (WDF) for the duration of 20 weeks. The body weight, whole body fat content, serum lipid profiles and liver function markers were examined monthly along with the assessment of liver histology for the development of NASH. In addition, the effects of obeticholic acid (OCA, 30 mg/kg, QD) on improvement of NASH progression in the model were evaluated.

**Results:**

Compared to normal control diet (CD), FATZO mice fed with WDF were heavier with higher body fat measured by qNMR, hypercholesterolemia and had progressive elevations in AST (~ 6 fold), ALT (~ 6 fold), liver over body weight (~ 2 fold) and liver triglyceride (TG) content (1.4–2.9 fold). Histological examination displayed evidence of NAFLD/NASH, including hepatic steatosis, lobular inflammation, ballooning and fibrosis in FATZO mice fed WDF. Treatment with OCA for 15 weeks in FATZO mice on WDF significantly alleviated hypercholesterolemia and elevation of AST/ALT, reduced liver weight and liver TG contents, attenuated hepatic ballooning, but did not affect body weight and blood TG levels.

**Conclusion:**

WDF fed FATZO mice represent a new model for the study of progressive NAFLD/NASH with concurrent metabolic dysregulation.

## Introduction

Non-alcoholic fatty liver disease (NAFLD) is an all-encompassing term used to describe the fatty liver environment in the absence of excessive alcohol consumption. It is estimated that 25% of the world’s general population meet the criteria for a diagnosis of NAFLD [1–3]. Metabolic disturbances are prominent risk factors for the development of NAFLD [3–10]. NAFLD with concurrent obesity, insulin resistance and hyperlipidemia is associated with an increased risk of progression of NAFLD to NASH. However, whether NAFLD develops prior to or as a result of metabolic dysregulation is unknown and debated [11–14].

The initial stage of NAFLD is characterized by the accumulation of ectopic fat in hepatocytes (steatosis). Steatosis is generally a benign, asymptomatic condition; however, with concurrent obesity/metabolic disturbances, steatosis can progress to non-alcoholic steatohepatitis (NASH) and in severe cases hepatocellular carcinoma (HCC) and liver failure [15]. Histologically NASH is characterized by hepatocellular ballooning, inflammation and increased liver fibrosis [16–18]. In the context of insulin resistance, obesity and dyslipidemia, an inflammatory response is initiated which is thought to be causative in the progression of NAFLD to more severe NASH [3, 6, 7, 19]. Unlike benign steatosis, NASH represents a significant health threat that progresses to fibrosis/cirrhosis in 10–28% of patients [12, 20–23]. Further progression from NASH to fibrosis/cirrhosis is highly predictive of mortality in these patients [24].

The study of human NAFLD and its progression is hampered by the slow (decades) development of disease as well as tools available for staging the disease [3]. While much research is ongoing to identify non-invasive tools for staging and reliable clinical biomarkers are not yet available, biopsy remains the gold standard. Thus attempts have been made to develop rodent models of fatty liver disease to aid in the investigation of the pathophysiological and morphological findings characteristic of NAFLD. As metabolic syndrome is the most prominent risk factor for the development of NAFLD in humans, the ideal animal model should develop NAFLD in the context of metabolic disease. Furthermore, the model should display histological characteristics such as steatosis, interlobular inflammation, hepatocellular ballooning, fibrosis and be susceptible to liver tumors seen in humans [12].

Over the last several years, investigators have taken different approaches to developing mouse models of NAFLD and NASH; methionine-choline deficient (MCD) diet [25], high fat diets with or without fructose in C57BL/6J and *ob/ob* mice [26–29] and the STAM model where 4dayold mice are given streptozotocin plus high fat diet [30, 31]. Initial attention has been placed on producing fibrosis as quickly as possible with MCD diet [27]. The mice on the MCD diet are not obese, actually losing significant body weight (30%), and are not insulin resistant or hyperlipidemia during disease progression [25]. The STAM model is characterized by type 1 diabetes induced with streptozotocin, rather than type 2 diabetes (T2D) on a high fat diet and produces fibrosis after 12 weeks on diet and eventually HCC [30, 31]. Streptozotocin, a known carcinogen, could exacerbate the development of HCC in this model. In C57BL/6 and Lep^*ob*^/Lep^*ob*^ models using high fat diets either alone or supplemented with fructose, investigators have observed NAFLD and NASH in the presence of insulin [26, 32–34].

The FATZO mouse was developed by crossing C57BL/6J and AKR/J mice that have a strong propensity to develop obesity when fed a high fat diet. Selective inbreeding of the offspring resulted in animals that have a strong propensity to develop many of the characteristics of “metabolic syndrome” early in life [35, 36]. Thus FATZO mice have a strong genetic pre-disposition towards obesity and develop insulin resistance, dyslipidemia, and obesity when fed a normal chow diet [35, 36].

Excess consumption of fructose has been shown to promote liver steatosis and fibrosis in humans [37–39] and normal rodents [40–42]. Therefore, we hypothesized that the pre-disposition for metabolic disruptions in the FATZO mice in conjunction with feeding of the WDF diet with fructose supplementation would generate a more translational model of NAFLD/NASH with characterization that resembles the progression of human disease. Thus, the aims of the present study were to examine 1) if FATZO mice fed WFD develop NAFLD/NASH similar to the pathologic changes in human; and 2) if obeticholic acid (OCA) treatment, one of the most advanced NASH specific drug in clinical trial, is able to improve liver function as well as morphological changes in the liver of FATZO mice fed WDF.

## Methods

### Animal studies

Male FATZO/Pco mice (*n* = 88) were bred and maintained at the Crown Bioscience facility (Indianapolis, IN). Animals were housed individually in open ventilated cages and fed control diet of Purina 5008 chow (LabDiet, St. Louis, MO) with distilled water ad libitum until study started. Room temperature was monitored and maintained at 20-26 °C with the light cycle set at 12 h (6:00–18:00). Mice at 8 weeks of age were randomized into different study groups based on body weight and serum ALT.

#### Effects of WDF on FATZO mice

In the first study for model characterization, animals were randomized into 2 groups fed with control diet (CD) (*n* = 32); or Western diet (D12079B, Research Diets, New Brunswick, NJ) + 5% fructose in the drinking water (WDF) (*n* = 32). Body weights were recorded weekly. Whole body fat content (%) was assessed using qNMR (EchoMRI-500; Houston, TX). Eight animals from each group were sacrificed every month for the duration of the study.

#### Effects of OCA in FATZO mice fed WDF

In the second study, all of FATZO mice were fed WDF at age 8 weeks for 8 weeks, then randomized into vehicle (*n* = 8) and OCA (30 mg/kg, p.o.QD, *n* = 8) groups for an additional 15 weeks on WDF. Previous studies have shown that to achieve significant differences between groups given the standard deviations of biochemical parameters, eight animals per group will be sufficient.

At the end of the studies, all mice were euthanized by neck dislocation, and blood samples and liver tissues were collected for examination. All animal experiments were approved by the Institutional Animal Care and Use Committee at Crown Bioscience – Indiana (IACUC protocol number: 2015–230).

### Biochemical measurements

Mice were fasted (6 h) prior to sacrifice and serum samples were obtained for clinical chemistry analysis including cholesterol, triglycerides, AST and ALT (AU480 clinical analyzer, Beckman-Coulter; Brea, CA).

Liver triglyceride content was analyzed from samples (~ 200 mg/animal) snap frozen in liquid nitrogen. Tissues were placed in Lysing Matrix D Tubes with distilled water in a 20% concentration (MP Biomedicals, Santa Anna, CA) and homogenized in a Fastprep-FP120 cell disrupter (Thermo Fisher Savant) for 30 s. Homogenates were kept cold and analyzed on a clinical analyzer (Beckman-Coulter AU480, Indianapolis, IN) within 30 min of preparation.

### Histology

#### Tissue processing

The liver tissues were fixed in 10% neutral buffered formalin (NBF) for 24 h followed by baths of standard concentrations of alcohol then xylene to prepare the tissues for paraffin embedding. After being embedded in paraffin and cooled, five-micron sections were cut and stained for routine H&E and Picro Sirius Red (PSR).

#### Whole slide digital imaging

A whole slide digital imaging system was used for imaging with the Aperio Scan Scope CS system (360 Park Center Drive, Vista, CA). The system imaged all slides at 20x. The scan time ranged from 1.5 to a maximum time of 2.25 min.

#### NAFLD activity score (NAS) and fibrosis scoring

The liver samples were evaluated by a trained general pathologist who is blind of different study groups using the NASH liver criteria for scoring [16–18]. Steatosis, lobular inflammation, hepatocyte balloon degeneration, fibrosis, NAS and the presence of NASH by pattern recognition were systematically assessed. Three representative areas per liver were assessed and the score of each parameter of individual animal was calculated by averaging scores from three representative areas [43].

### Statistics

Treatment effects of WDF were compared to CD over the time points using One-Way or Repeat Measures ANOVA with multiple comparison t-test using Prism (GraphPad, version 7.01). Statistical differences were denoted as *p* < 0.05. All values are reported as Mean ± SEM; unless noted otherwise.

## Results

### WDF exacerbated metabolic disorders, impaired liver function and caused histological changes resembling NAFLD/NASH in FATZO mice

**Fig. 1:**
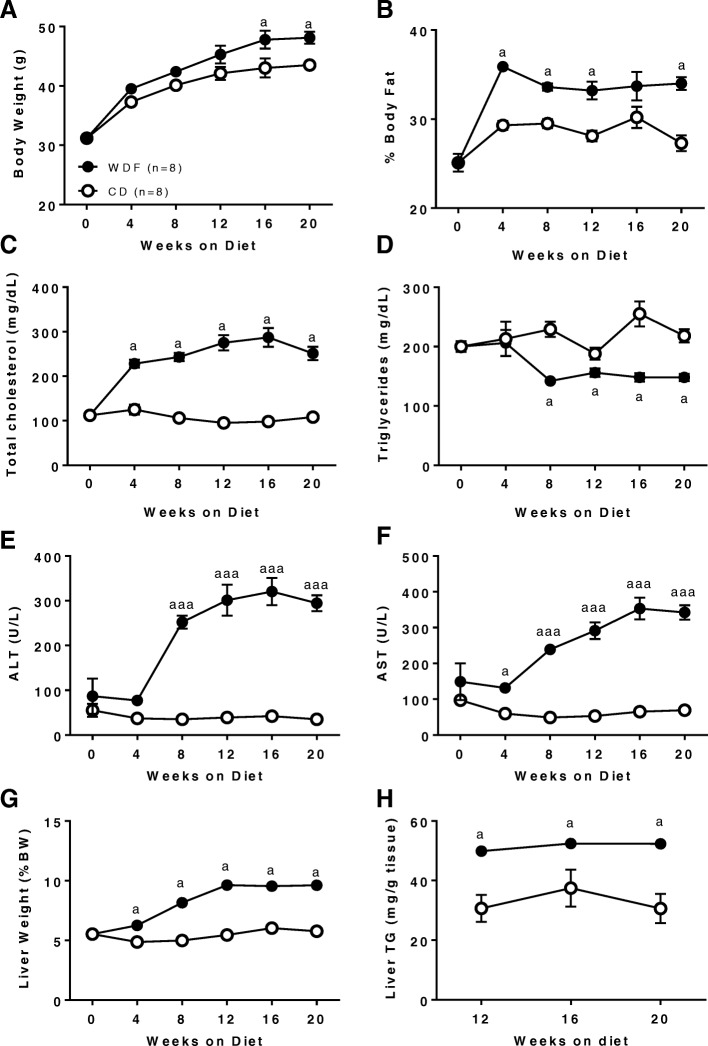
Development of NAFLD/NASH in FATZO mice fed with CD or WDF diet. (**a**) Body weight, (**b**) body fat, (**c**) total cholesterol, (**d**) triglyceride, (**e**) ALT, (**f**) AST, (**g**) liver weight, and (**h**) hepatic triglyceride content in FATZO mice fed with CD or WDF for 20 weeks. Data were presented as mean ± SEM. ^a^*p* < 0.05, ^aaa^*p* < 0.005 vs vehicle controls using two-way ANOVA with Dunnett’s post-hoc comparison

The FATZO mice fed WDF showed a significantly greater increase in body weight (Fig. 1a), associated with a significant increase in body fat compared to the age-matched CD fed mice (Fig. 1b). Blood cholesterol levels were almost 2.5 times higher in WDF group than CD controls after 4 weeks on diet and the levels were consistently higher in WDF group throughout the diet induction period (Fig. 1c), though triglyceride levels were slightly lower (Fig. 1d) in the WDF group.

Metabolic stress on the livers of FATZO mice fed WDF caused a significant elevation in the liver enzymes, with evidence of almost 6 and 4 fold higher in ALT (Fig. 1e) and AST (Fig. 1f) levels respectively over the 20 weeks of diet exposure compared to that in CD fed mice. The liver over body weight significantly increased over time (Fig. 1g), accompanied by a significantly higher liver TG content measured at weeks 12–20 from mice fed WDF compared to that of CD (Fig. 1h).

**Fig. 2:**
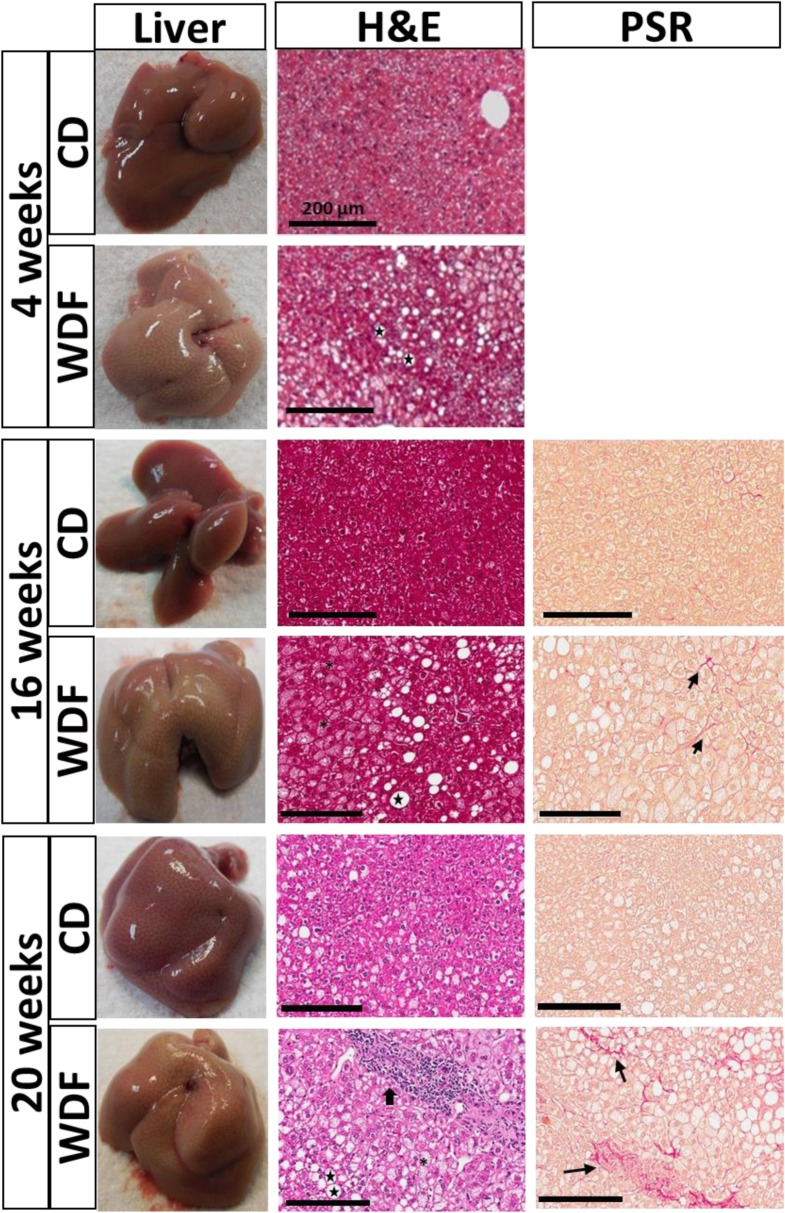
Histological evidence of NAFLD/NASH in FATZO mice fed WDF. Representative images of H&E and Picro Sirius Red (PSR) staining of livers removed from FATZO mice fed WDF or CD for 4, 16 and 20 weeks. Denotes steatosis, denotes ballooning, denotes lobular inflammation and denotes fibrosis

WDF fed FATZO mice developed fatty liver characterized by progressive steatosis, hepatocellular ballooning, lobular inflammation and the early stages of fibrosis. During the early progression of NAFLD, the livers from WDF fed FATZO mice were very pale in color upon necropsy compared to that of CD fed mice (Fig. 2). H&E staining demonstrated pan lobular steatosis with ballooning as early as 4 weeks on WDF compared to CD diet. Over time, FATZO mice exhibited a progressive worsening of NAFLD. At each time point, the livers of WDF fed mice were paler in color than the corresponding CD fed ones. Significant histological changes indicative of NASH (steatosis, hepatocellular ballooning, lobular inflammation and mild fibrosis) were seen in the liver sections from the mice on WDF for 16 weeks (Fig. 2).

**Fig. 3:**
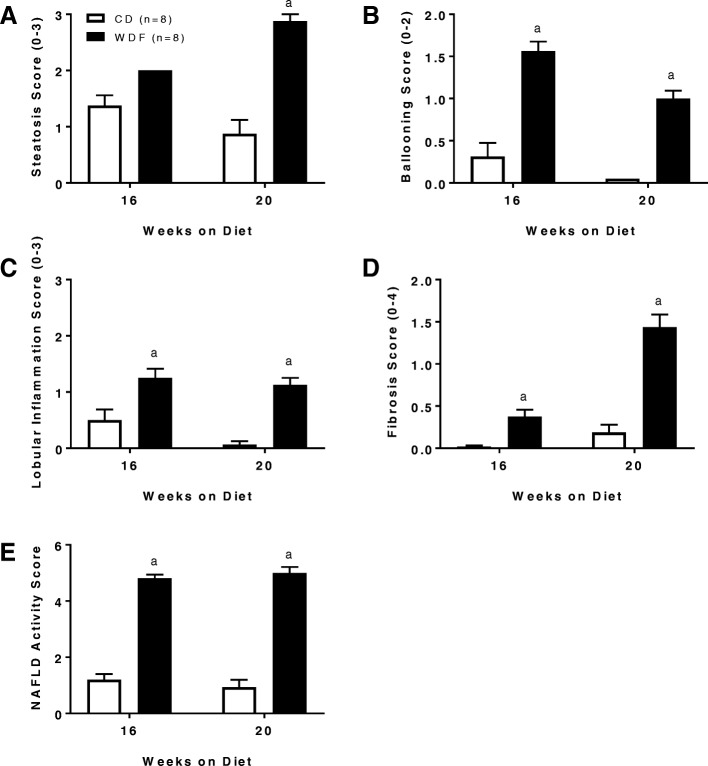
NASH scoring of the liver from WDF or CD fed FATZO mice for 16 and 20 weeks. (**a**) Steatosis, (**b**) hepatic ballooning, (**c**) lobular inflammation, (**d**) Fibrosis and (**e**) NAS scores. Data were presented as mean ± SEM. ^a^*p* < 0.05vs vehicle controls using two-way ANOVA with Dunnett’s post-hoc comparison

When sections were assessed for individual NASH activity scores, the livers from WDF fed mice exhibited significantly higher scores for steatosis (Fig. 3a), hepatocellular ballooning (Fig. 3b), lobular inflammation (Fig. 3c) and fibrosis (Fig. 3d) when compared to the corresponding livers from the CD fed mice. When measured by a composite NAFLD activity score (NAS), the livers from WDF fed mice demonstrated significantly more pathological findings when compared to the livers from CD fed mice (Fig. 3E).

### OCA improved liver function and hepatic ballooning in FATZO mice fed WDF

**Fig. 4:**
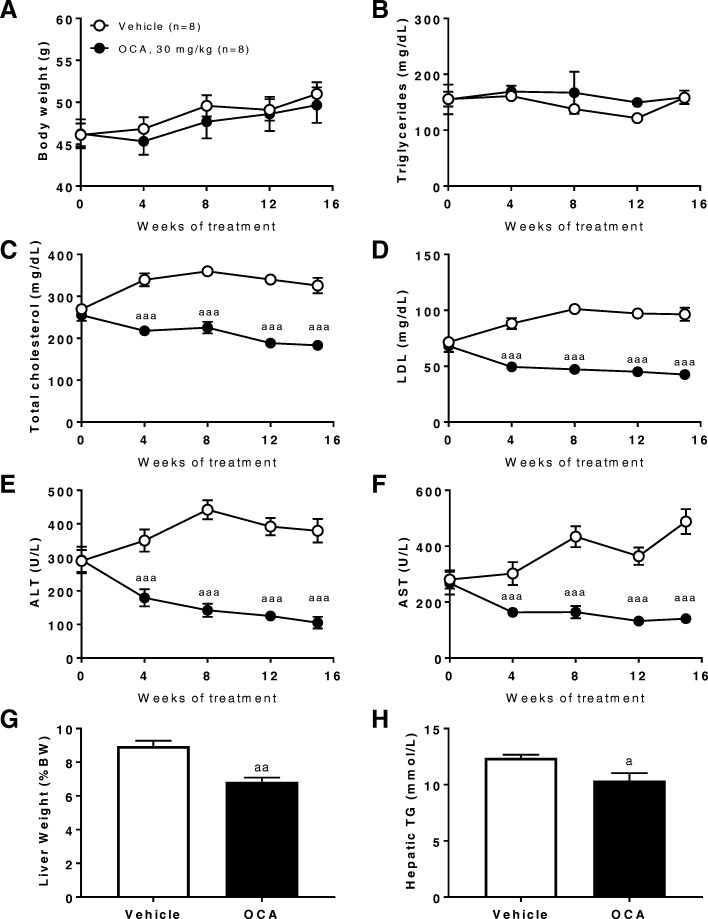
OCA treatment improves liver function and lipid metabolism in FATZO mice fed WDF. (**a**) Body weight, (**b**) blood triglyceride, (**c**) total cholesterol, (**d**) LDL, (**e**) ALT, (**f**) AST, (**g**) liver weight, and (**h**) hepatic triglyceride content in WDF fed FATZO mice treated with vehicle or OCA (30 mg/kg, QD). Data were presented as mean ± SEM. ^a^*p* < 0.05, ^aa^*p* < 0.01, ^aaa^*p* < 0.005vs vehicle controls using two-way ANOVA with Dunnett’s post hoc comparison (**a**-**f**) or unpaired student t-test (**g**, **h**)

Treatment of OCA (30 mg/kg, QD) in FATZO mice on WDF from 8 weeks on diet for additional 15 weeks had no impact on body weight (Fig. 4a) or blood TG levels (Fig. 4b). In contrast, OCA treatment alleviated the elevation of blood total cholesterol (Fig. 4c) and LDL (Fig. 4d), resulting in significantly lower levels compared to vehicle controls. In addition, improvement in liver function could be seen as early as 4 weeks after OCA treatment, as blood ALT (Fig. 4e) and AST (Fig. 4f) levels in the OCA treatment group were dramatically lower than its own pretreatment baseline as well as the CD fed mice. After treatment with OCA for 15 weeks, liver over body weight (Fig. 4g) and hepatic TG levels decreased significantly compared to the vehicle controls (Fig. 4h).

**Fig. 5:**
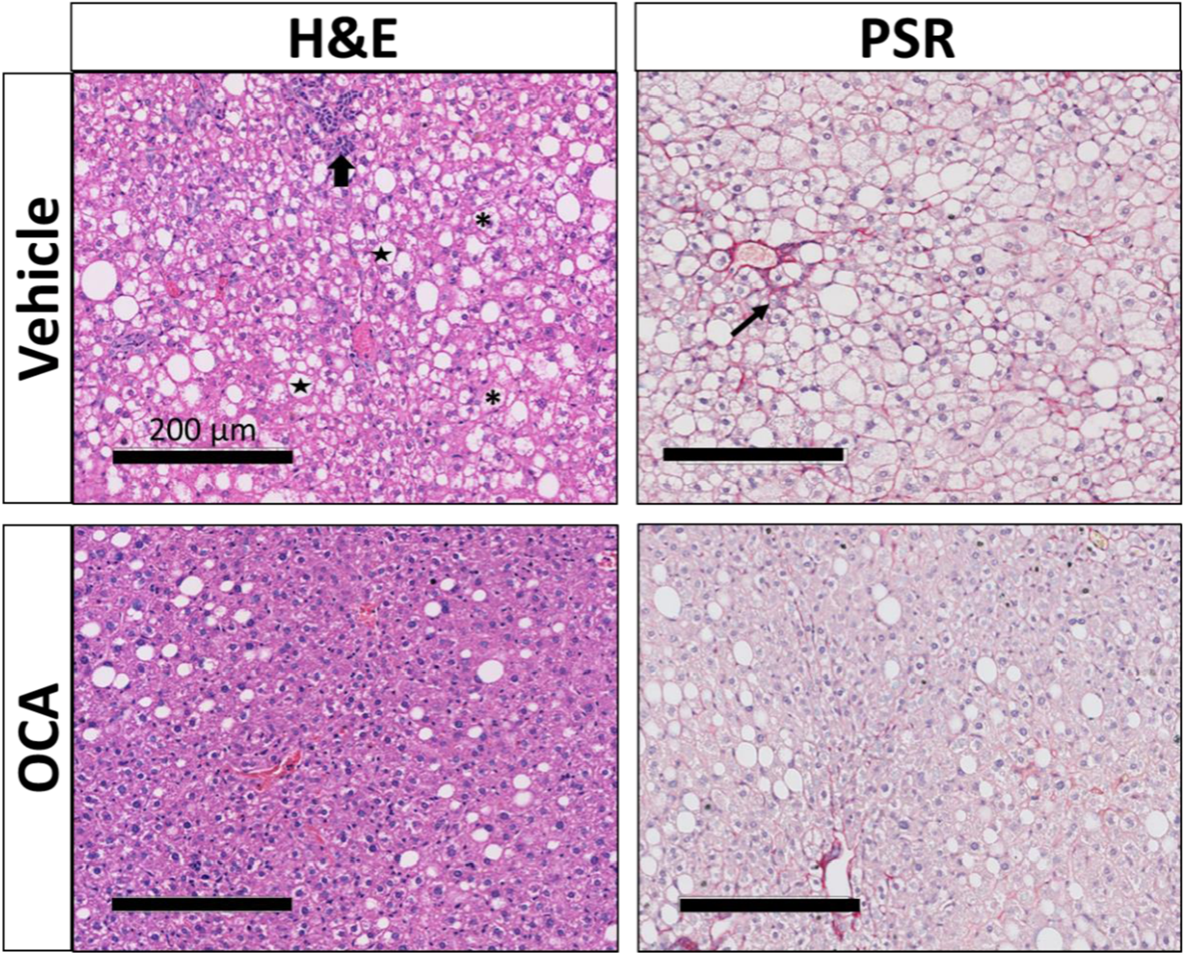
OCA treatment improves hepatic ballooning in NASH FATZO mice. Representative images of H&E and Picro Sirius Red (PSR) staining of the livers removed from NAFLD/NASH FATZO mice treated with OCA or vehicle for 15 weeks

**Fig. 6:**
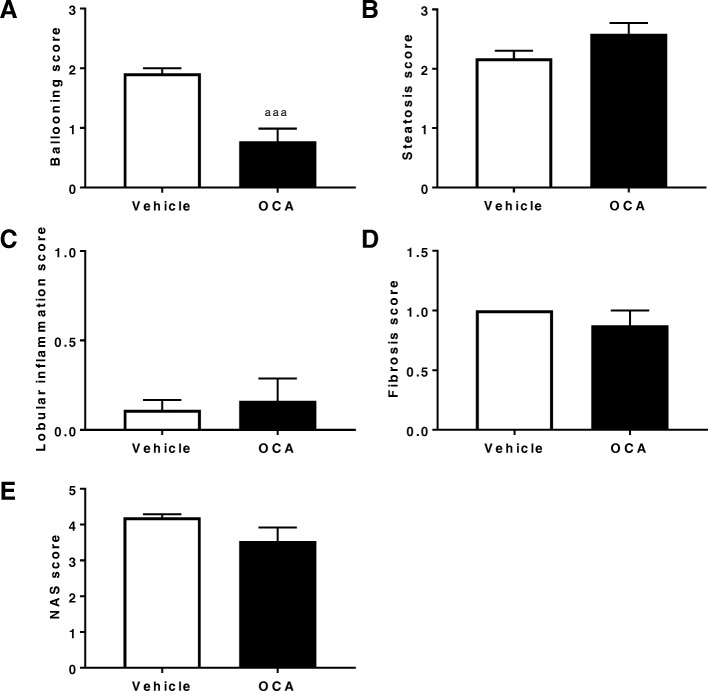
Histological improvement of WDF fed FATZO mice treated with OCA. (**a**) Hepatic ballooning, (**b**) Steatosis, (**c**) Lobular inflammation, (**d**) Fibrosis and (**e**) NAS score. Data were presented as mean ± SEM. ^aaa^*p* < 0.005 vs vehicle controls, using unpaired student t-test

When liver histology was evaluated (Fig. 5), OCA treatment tended to improve NAS score (Fig. 6e) with significant alleviation in numbers of foci showing hepatic ballooning (Fig. 6b). The changes in other components of NAS score, such as steatosis (Fig. 6a), lobular inflammation (Fig. 6c), and fibrosis (Fig. 6d) were not obvious.

## Discussion

Currently, the global hypothesis for the pathogenesis of NASH is the “multi-hit hypothesis,” with metabolic syndrome playing a major role, due to insulin resistance followed by pro-inflammatory processes. Unlike monogenic leptin deficient *ob/ob* or *db/db* mice, the FATZO mice area polygenic model of obesity and type 2 diabetes when fed regular rodent diet, with an intact leptin pathway, thereby making it more translatable to the human disease [35, 36]. The goal of the present investigation was to determine if the FATZO mice, which inherently develop metabolic syndrome and type 2 diabetes, would develop NAFLD and NASH when fed a western diet supplemented with fructose.

In recent reviews [12, 44], the murine models that most closely resembled the human disease, were those that used high fat diets supplemented with fructose. These diets best simulate the high prevalence of high fat food and corn syrup sweetened beverages in the Western diet. Fructose has been shown to enhance the development of NAFLD and NASH without [29], or with fibrosis in C57BL/6 [26, 28, 34, 45], *ob/ob* [28, 34] and DIAMOND™ [32] mice on high fat diets.

In the present investigation, FATZO mice fed WDF diet developed NAFLD and NASH with progressive steatosis, ballooning, inflammation and mild fibrosis over 20 weeks when compared to the CD. In the plasma, increases in the liver enzymes, ALT/AST, and cholesterol were observed in FATZO mice on WDF diet for as early as 4 weeks and remained significantly higher compared to the values from the mice on CD over 20 weeks. Plasma triglycerides were not elevated in WDF fed animals when compared to CD, which is consistent with reports in the *ob/ob* NASH models [28, 34]. However, liver triglycerides were elevated 1.4–2.9 folds at 12, 16 and 20 weeks in mice fed WDF compared to CD. On gross necropsy, the livers from WDF fed mice were pale in color and had significantly higher liver over body weight ratios when compared to the corresponding CD fed group. Histologically, the livers from WDF fed mice presented steatosis as early as 4 weeks on diet which progressed to steatohepatitis characterized by balloon degeneration, lobular inflammation and fibrosis. The composite NAS score in the FATZO mice fed WDF was equivalent to “~ 5” at 16 and 20 weeks on diet; indicative of “definitive” NASH [17, 18]. Mild fibrosis was observed as early as 16 weeks on diet in 50% of WDF fed mice and progressed to 100% of animals demonstrating moderate fibrosis scores of 1.5 at 20 weeks.

Recently, Machado et al. reported that the models with Western diet developed a more common and relatively non-progressive subtype of NASH, whereas MCD diet model developed a less common and more rapidly progressive/aggressive NASH subtype [25]. The main differences between the 2 models are significant in regards to which more closely mimics the human condition. Western diet fed animals are obese, insulin resistant and hyperlipidemia whereas the MCD diet fed animals had weight loss, but are not insulin resistant or hyperlipidemia [25]. FATZO mice on WDF benefit with progressive NASH phenotypes, increased body weight and hyperlipidemia. More recently, when 22%fructose and trans-fat are added to the Western diet, *ob/ob* mice develop steatosis, lobular inflammation and mild fibrosis similar to FATZO mice on WDF [28, 34]. However, compared to FATZO mice on the WDF, the hepatic ballooning was not present in the *ob/ob* mice on Western diet via the same length of diet induction [28]. More importantly, the use of *ob/ob* mice with intrinsic leptin deficiency in the metabolic disease should always be cautious, as the involvement of leptin in multiple metabolic signaling pathways should not be overlooked. As a result, the appearance of NASH phenotypes in the models with gene mutations might not fully reflect the actual disease pathogenesis, which might create concerns for the model to be used in testing anti-NASH therapy. Compared to other rodent NASH models where animals with intrinsic gene mutation (eg: *ob/ob* mice) or chemical induction, FATZO mice have more physiological relevance to human patients with metabolic syndromes, while maintaining critical biochemical and histopathological changes of phenotypes representative of NASH by applying the common risk factor of inducing NASH such as fructose.

The diet induced obesity (DIO) model in C57BL/6J mice also present liver TG accumulation and hepatic steatoses, however, unlike the FATZO mice, C57BL/6J mice are not diabetic, and develop less severe liver histopathologic changes.

Multiple drugs are in the development stage for treatment of NASH. Obeticholic acid (OCA) is a semi-synthetic bile acid that acts on the nuclear farnesoid X receptor (FXR) which is expressed predominantly in the liver, kidney and intestine to regulate bile acid homeostasis, hepatic lipid metabolism and immune function [46, 47]. It was originally developed for the treatment of primary biliary cholangitis [48] and is currently in the most advanced stage being tested for NASH in several clinical trials with evidence of significant alleviation of plasma liver ALT and AST levels and mild improvement in steatosis, hepatic ballooning, lobular inflammation and fibrosis [49]. In pre-clinical rodent studies, OCA has been shown to reduce hepatic lipid accumulation, liver enzyme activities, steatosis and fibrosis, though the models and dosing regimen selected might largely affect the final manifestation of the drug efficacy [50, 51]. FATZO mice which possess aberrant lipid metabolism develop advanced NASH phenotypes upon WD feeding. The development of the phenotypes was largely accelerated by the impact of fructose on hepatic lipid metabolism including lipogenesis and reduced fatty acid oxidation. The treatment of FATZO mice with OCA is likely to reduce such impact by inhibiting lipogenesis and fatty acid synthesis. Indeed, in the present study in FATZO mice on WFD for 8 weeks, OCA treatment for 15 weeks significantly reduced plasma ALT and AST levels almost to baseline values before WDF induction (Fig. 4e and e). This result is consistent with the report from NASH patients treated with OCA [49]. In addition, treatment of OCA for 8 weeks appeared to be more efficacious in reducing liver enzymes in FATZO fed WDF compared to *ob/ob* mice fed with AMLN diet, where impacts of OCA on plasma liver enzymes in the latter model were minimal [51]. Moreover, OCA treatment improved hepatic ballooning leading to overall reduction in NAS score and increased the numbers of animals with absence of fibrosis in WDF fed FATZO mice (Fig. 5 and 6). The data suggested that the leading anti-NASH treatment OCA can improve NASH phenotypes in FATZO mice fed with WDF similar as seen in human patients. More importantly, some of the known effects of OCA such as reducing liver enzyme ALT in NASH patients which were not shown in ob/ob mice were also evident in FATZO model, suggesting more clinical relevance of the model. Therefore, FATZO mice fed with WDF develop NAFLD/NASH phenotypes in a time frame that might be suitable for testing anti-NASH drug intervention.

## Conclusion

In the present investigation, the polygenic FATZO mouse model of obesity and type 2 diabetes with an intact leptin pathway diet developed progressive NAFLD/NASH similar to humans when fed with WDF. The FATZO WDF model of NAFLD/NASH represents a new and improved scientific tool for the advancement of NAFLD/NASH research which is potentially more translatable to human disease than many other models.

Based on the results of this study, other previously published studies and for a better description of model, the common name of this model will be subsequently changed from FATZO/Pco to MS-NASH/PcoJ and as such will be marketed through Jackson Labs.

## Abbreviations

ALT: Alanine aminotransferase
AST: Aspartate transaminase
BW: Body weight
CD: Control diet
FXR: Farnesoid X nuclear receptor
H&E: Hematoxylin and eosin
HCC: Hepatocellular carcinoma
LDL: Low density lipoprotein
MCD: Methionine-choline deficient
NAFLD: Non-alcoholic fatty liver disease
NAS: NAFLD activity score
NASH: Non-alcoholic steatohepatitis
NBF: Neutral buffered formalin
NMR: Nuclear magnetic resonance
OCA: Obeticholic acid
PSR: Picrosirius red
QD: Quaque die
T2D: Type 2 diabetes
TC: Total cholesterol
WDF: Western diet with fructose
